# Achievements in mental health outcome measurement in Australia: Reflections on progress made by the Australian Mental Health Outcomes and Classification Network (AMHOCN)

**DOI:** 10.1186/1752-4458-6-4

**Published:** 2012-05-28

**Authors:** Philip Burgess, Tim Coombs, Adam Clarke, Rosemary Dickson, Jane Pirkis

**Affiliations:** 1 School of Population Health, University of Queensland, Locked Bag 500, Sumner Park BC, Queensland, 4077, Brisbane, Australia; 2 Australian Mental Health Outcomes and Classification Network, New South Wales Institute of Psychiatry, Parramatta, Parramatta, Australia; 3 Strategic Data Pty Ltd, Melbourne, Australia; 4 Melbourne School of Population Health, University of Melbourne, Melbourne, Australia

## Abstract

**Background:**

Australia’s National Mental Health Strategy has emphasised the quality, effectiveness and efficiency of services, and has promoted the collection of outcomes and casemix data as a means of monitoring these. All public sector mental health services across Australia now routinely report outcomes and casemix data. Since late-2003, the Australian Mental Health Outcomes and Classification Network (AMHOCN) has received, processed, analysed and reported on outcome data at a national level, and played a training and service development role. This paper documents the history of AMHOCN’s activities and achievements, with a view to providing lessons for others embarking on similar exercises.

**Method:**

We conducted a desktop review of relevant documents to summarise the history of AMHOCN.

**Results:**

AMHOCN has operated within a framework that has provided an overarching structure to guide its activities but has been flexible enough to allow it to respond to changing priorities. With no precedents to draw upon, it has undertaken activities in an iterative fashion with an element of ‘trial and error’. It has taken a multi-pronged approach to ensuring that data are of high quality: developing innovative technical solutions; fostering ‘information literacy’; maximising the clinical utility of data at a local level; and producing reports that are meaningful to a range of audiences.

**Conclusion:**

AMHOCN’s efforts have contributed to routine outcome measurement gaining a firm foothold in Australia’s public sector mental health services.

## Introduction

Australia’s National Mental Health Strategy emphasises the importance of improving the quality and effectiveness of publicly-funded mental health services, promoting routine consumer outcome measurement as a vehicle to monitor such improvements [1-5]. The Strategy also stresses the need to examine service efficiency, advocating further development of casemix classifications to identify the resourcing required to care for ‘typical’ groups of consumers.

Initially, the Strategy focused on identifying and trialling a set of measures that could further the routine outcome measurement and casemix development agendas [6-9]. In the late 1990s, once the measures were settled upon, attention shifted to establishing the necessary infrastructure and resources to support routine data collection using these measures. A statement of information development priorities was prepared [10], and the Australian Government signed agreements with each of the state/territory governments which bound the latter to routinely collect and submit relevant data. The agreements committed the Australian Government to: provide funding to states/territories to develop appropriate information systems; support efforts to equip the clinical workforce for outcome measurement (e.g., by developing nationally-consistent training resources and providing a clearing house to encourage the use of outcomes and casemix data); establish three national mental health outcomes expert groups (adult, child/adolescent and older persons) to advise on issues of implementation and data usage; and put in place arrangements to receive, process, analyse and report on outcome data at a national level [11].

A protocol to guide the collection of the Mental Health National Outcomes and Casemix Collection (MH-NOCC) was released in 2002 [12], and has since been updated [13]. The MH-NOCC protocol requires that clinician-rated and consumer-rated measures are administered at particular ‘collection occasions’ (admission, review and discharge) during each ‘episode of care’ (a more or less continuous period of contact between a consumer and a mental health service organisation that occurs within the one inpatient or community mental health service setting. The measures vary according to the age group targeted by the particular service setting, and are summarised in Table 1.

**Table 1:** Administration of measures according to the MH-NOCC protocol

	**Inpatient**			**Community residential**			**Ambulatory**		
	**Admission**	**Review**	**Discharge**	**Admission**	**Review**	**Discharge**	**Admission**	**Review**	**Discharge**
*Children and Adolescents*									
HoNOSCA	●	●	●	●	●	●	●	●	●
CGAS	●	●	-	●	●	-	●	●	-
FIHS	-	●	●	-	●	●	-	●	●
Principal and Additional Diagnoses	-	●	●	-	●	●	-	●	●
Mental Health Legal Status	-	●	●	-	●	●	-	●	●
*Adults*									
HoNOS	●	●	●	●	●	●	●	●	●
LSP-16	-	-	-	●	●	●	-	●	●
Consumer self-report	-	-	-	●	●	●	●	●	●
Principal and Additional Diagnoses	-	●	●	-	●	●	-	●	●
Focus of Care	-	-	-	-	-	-	-	●	●
Mental Health Legal Status	-	●	●	-	●	●	-	●	●
*Older persons*									
HoNOS 65+	●	●	●	●	●	●	●	●	●
LSP-16	-	-	-	●	●	●	-	●	●
RUG-ADL	●	●	-	●	●	-	-	-	-
Consumer self-report	-	-	-	●	●	●	●	●	●
Principal and Additional Diagnoses	-	●	●	-	●	●	-	●	●
Focus of Care	-	-	-	-	-	-	-	●	●
Mental Health Legal Status	-	●	●	-	●	●	-	●	●

**LEGEND:** HoNOS: Health of the Nation Outcome Scales (Clinician-rated); LSP-16: Life Skills Profile 16 (Clinician-rated); MHI: Mental Health Inventory (Consumer-rated); BASIS-32: Behaviour and Symptom Identification Scale (Consumer-rated); K-10+: Kessler 10 Plus (Consumer-rated); HoNOSCA: Health of the Nation Outcome Scales for Children and Adolescents (Clinician-rated); CGAS: Children’s Global Assessment Scale (Clinician-rated); SDQ: Strengths and Difficulties Questionnaire (Consumer/Parent-rated); HoNOS65+: Health of the Nation Outcome Scales 65+ (Clinician-rated); RUG-ADL: Resource Utilisation Groups – Activities of Daily Living (Clinician-rated).

Table adapted from Department of Health and Ageing [12].

In late 2003, we were contracted by the Australian Government’s Department of Health and Ageing to form a consortium known as the Australian Mental Health Outcomes and Classification Network (AMHOCN), to undertake the tasks mentioned above: data management; training and service development; and analysis and reporting. Our work program has been shaped by the key mental health service system question posed by Leginski and US colleagues in 1989: ‘Who receives what services from whom, at what cost, and with what effect?’ [14].

When we began, we conducted a series of stakeholder consultations to gauge national progress with the ‘roll-out’ of MH-NOCC data collection. Each state/territory was asked to invite key players with an interest in collecting or using outcome data to participate in these consultations. States/territories were told that these stakeholders might include technical personnel, consumers, carers, clinicians and policy-makers, but the exact list of invitees was left to their discretion. The consultations revealed that all states/territories had begun to routinely collect and submit de-identified, consumer-level outcome data broadly in accordance with the MH-NOCC protocol, but there was considerable variability regarding data coverage, completeness and compliance [15,16]. The consultations also indicated that the casemix development agenda faced particular difficulties. Progressing this agenda required linkage between the MH-NOCC dataset and other national datasets that contain information from which resource use could be estimated (e.g., the Admitted Patients Mental Health Minimum Data Set, the National Minimum Data Set for Community Mental Health Care, and the Residential Mental Health Minimum Data Set), and, in most cases, different systems of unique identifiers precluded this linkage [15,16].

Over time, the quantity and quality of outcome data has improved significantly. More importantly, clinicians and consumers have come to value outcome data, albeit to varying degrees [17,18]. In addition, there are examples of outcome data being used in local service settings to guide clinical decision-making, engage consumers in treatment, foster a collaborative approach to care planning and goal setting, review consumers’ progress with treatment, inform questions about consumers’ eligibility for given programs, assist with discharge planning, improve the evidence-base on which services are founded, and evaluate particular models of service delivery [19,20]. Casemix development has not proceeded at the same pace as routine outcome measurement, but some progress has been made.

Australia’s achievements in this area reflect significant investments by all jurisdictions. We have been privileged to have been involved at the outset and have had a unique opportunity to shape that evolving agenda. In 2010, the Department of Health and Ageing commissioned Healthcare Management Advisors (HMA) to conduct an independent review of AMHOCN which gave us the opportunity to reflect on our performance and achievements since our establishment in late 2003. This paper documents the history of AMHOCN and its role supporting Australia’s public sector mental health services to monitor their quality, effectiveness and efficiency. The paper focuses mainly on our efforts with respect to routine outcome measurement because this is where we have made the greatest gains; some mention is made of our casemix development work, but the external data linkage issues have hampered our progress in this area.

## Method

We drew on a number of documents to summarise the history of AMHOCN. These included: our regular progress reports to the Australian Government; topic-specific reports and presentations that we have prepared for particular audiences; and peer-reviewed journal articles. Many of these materials are publically available on the AMHOCN website – http://amhocn.org The history is presented thematically rather than chronologically, partly because many of our activities have been evolving and/or ongoing, and partly because we think that this approach highlights the learnings so far.

## Results

### Establishing the appropriate infrastructure and mode of operation

In late 2003, following a competitive tender process, the Australian Government contracted with our respective organisations to deliver services under AMHOCN: Strategic Data (AC) to develop and maintain a data bureau; the University of Queensland (PB/JP) to manage and lead the analysis and reporting functions; and the New South Wales Institute of Psychiatry (TC) to take responsibility for training and service development. It became apparent quite early that there were significant interdependencies among the three components as well as challenges for the consortium being geographically dispersed. These interdependencies required that we worked closely together, so we developed a collaborative work plan to progress the outcome measurement agenda (and have since done so annually). We also appointed an AMHOCN network coordinator (RD) to co-ordinate activities across the three components. In late-2004, for a brief time, the Australian Government also appointed a state/territory liaison manager to assist state/territory health authorities to supply comprehensive data and to assist AMHOCN to provide reports that could guide service quality improvement. After 12 months, this role was subsumed into the work of the three components of AMHOCN.

These arrangements have enabled us to collaborate on various issues. For example, there have often been circumstances where the analysis and reporting component has identified particular data anomalies, the data bureau has worked with states/territories to find solutions, and the training and service development component has communicated these solutions to the field. Similarly, there have been a number of occasions where the training and service development component has identified stakeholder information needs, the analysis and reporting component has translated these into standard reports and other products, and the data bureau has automated and/or individualised these outputs.

### Locating AMHOCN within a broader policy framework

From the outset, AMHOCN has been located within a broad policy framework that has guided our co-ordinated national approach. We have reported to health ministers via the quarterly meetings of the Mental Health Information Strategy Subcommittee (MHISS), which makes recommendations on the information requirements of the National Mental Health Strategy. MHISS reports to the Mental Health Standing Committee (MHSC), whose role is to monitor the implementation of Australia’s National Mental Health Strategy and to support cross-state/territory communication and information exchange to improve outcomes from these policy reforms. The National Mental Health Performance Subcommittee (NMHPSC) is a subcommittee of the MHISS, which oversees the development and implementation of the national performance measurement framework for mental health services to support benchmarking for mental health service improvement, and provide national information on mental health system performance. Throughout its tenure, AMHOCN has regularly provided advice to the MHISS and the NMHPSC, and, through them, to the MHSC. The MHSC reports to Australia’s health ministers (previously the Australian Health Ministers Conference, or AHMC, now the Standing Committee on Health, or SCoH) via the Health Policy Priorities Principal Committee (HPPPC) and the Australian Health Ministers' Advisory Council (AHMAC).

Our relationship with the expert groups is worthy of mention here. Three expert groups (adult, child/adolescent and older persons) were appointed in 2004 to provide advice on routine outcome measurement from the perspective of clinicians, consumers and carers. Their structure reflected the way in which mental health services are organised in Australia, and the way in which MH-NOCC data are collected. They existed in their original form until 2009, when MHISS reviewed them and recommended that they be re-structured to encourage common solutions across the three program streams. The result was a new structure comprising a national mental health information development expert advisory panel, and four program-specific panels (adult, child/adolescent, older persons, forensic). The expert groups and panels have advised government about the implementation and use of routine outcome measurement (and other mental health information development initiatives). We have supported them in this role (e.g., providing them with profiles of scores on relevant measures). The expert groups and panels have helped us in our role, offering us guidance on our activities and providing a conduit for information dissemination.

The broader policy context within which we sit has guided our work and enabled us to influence mental health information developments. For example, calls to develop and operationalise key performance indicators for the Fourth National Mental Health Plan [5] and the Council of Australian Governments National Action Plan on Mental Health [21] led us to articulate ways of measuring effectiveness, using the Health of the Nation Outcome Scales (HoNOS) family of measures. Similarly, the Fourth National Mental Health Plan’s [5] commitment to accountability resulted in our undertaking a program of work around public reporting of data against national standards. We collaborated with relevant parties to review the literature on public reporting, and helped develop recommendations about the form such public reporting might take and the governance arrangements that might underpin it [22].

We have been able to identify and capitalise on local successes and find solutions to common problems. Being part of the formal structure has also given us the imprimatur of the National Mental Health Strategy, without which it would have been difficult to embed outcome measurement within routine practice.

### Facilitating multi-way communication about outcome measurement

As mentioned above, one of our earliest activities – conducted in 2004 – was a series of consultations with stakeholders from all states/territories [15]. In total, 123 individuals attended our consultation sessions (policy-makers and technical personnel from central mental health units and mainstream health information sections, service managers, clinicians, individuals responsible for reporting routine outcome measurement at a site level, consumers and carers, and expert group members). These stakeholders helped us to understand that there was considerable variability in the ‘state of play’ with respect to routine outcome measurement at that point, which guided our tailored approach to supporting these endeavours thereafter.

We have continued to seek guidance from and provide advice to numerous organisations and individuals (e.g., the expert groups and panels), formalising this process in a communication strategy. The resultant iterative feedback loop has been a feature of our way of operating, and has encouraged mutual sharing of knowledge about outcome measurement within the broader field. Sometimes we have sought feedback on particular issues from stakeholders, and sometimes they have instigated communication themselves. Sometimes we have undertaken a formal thematic analysis of stakeholder comments (e.g., in our original stakeholder consultations), but more commonly we have considered stakeholders’ opinions in a more informal manner.

The training and service development component has facilitated events that have brought together service managers, clinicians, consumers and carers to showcase the use of outcome measures. AMHOCN has held forums and workshops in all states/territories every year since its inception, and, in conjunction with Te Pou, New Zealand’s National Centre of Mental Health Research, Information and Workforce Development. Te Pou has been funded by the Ministry of Health to create a ‘mental health hub’ for New Zealand. A key part of its work program involves supporting the implementation and use of routine outcome measurement in New Zealand’s mental health services. AMHOCN and Te Pou have collaborated on the organisation of three Australasian Mental Health Outcomes Conferences (in Wellington in 2007, in Melbourne in 2008, and in Auckland in 2010). These events have highlighted exemplary practice in using outcome measurement to monitor service quality.

Early on we created a website (http://mhnocc.org/) to support the collection of outcome data. This site was not well utilised; user feedback indicated it was too narrow in its focus and difficult to navigate. It was also time-consuming for us to maintain because it hosted forums which we moderated. Consequently, we developed an alternative site (http://amhocn.org/) which emphasises the broader information development enterprise and is more user-friendly. This provides access to all of our materials, and contains online forums that are more targeted than the original ones. These forums have faced some challenges because of the variation across states/territories in terms of information technology infrastructure and staff access, but have facilitated some fruitful conversations between stakeholders. Statistics for the 4-month period from the beginning of November 2010 to the end of February 2011 provide an indication of the levels of uptake (3,525 hits on the home page; 1,282 on the online training page; 741 on the ‘useful resources’ page; 726 on the forums and workshops page; and 608 on the training resources page). We regard these levels as high, given the targeted nature of the material.

In addition to facilitating general information exchange via face-to-face events and the web-based forum, we have held regular meetings for specific groups. For example, we have hosted annual national trainers’ meetings since 2006. These have enabled those responsible for training clinicians in routine outcome measurement to support each other, share materials, and identify common needs. In a similar vein, we have held meetings for individuals who analyse and report on MH-NOCC data at a local level. Again, these forums have enabled participants to learn from each other, particularly with respect to technical issues (e.g., dealing with missing data, calculating change scores). The trainers’ and data analysts’ meetings have helped to foster a nationally consistent approach to salient aspects of routine outcome measurement.

Our communication strategy has also involved highlighting achievements with respect to routine outcome measurement. We have regularly presented at national and international mental health and general health conferences, and have published peer-reviewed articles in academic journals. We have also published a range of discussion papers and reports on particular conceptual and technical issues (available via our website).

### Equipping the mental health workforce to collect and use outcome data

Our efforts to equip the mental health workforce to collect and use outcome data began with the development of basic training materials that described the administration of the MH-NOCC suite of measures. These materials built on a range of pre-existing documents, including manuals [23-25], glossaries [26], policy documents and technical reports [27-29]. We aimed to make the new materials nationally consistent, but this presented challenges. Although there was a national data collection protocol, there was no agreement as to which consumer-rated measure should be used and the protocol had been modified in some states/territories (e.g., NSW had elected to collect the Kessler-10 ( K-10) in inpatient settings, despite the national protocol only requiring collection in community settings) [15]. Our solution was to develop a core set of training materials (structured around age group and service setting) that could be modified to meet local needs.

As time went on, stakeholders indicated that a shift in emphasis was needed. There were calls for routine outcome measurement to have greater clinical utility at a local level, so, in collaboration with Barwon Health, we developed a set of training materials that focused on using the consumer-rated outcome measures to promote clinician-consumer dialogue and guide clinical decisions. Clinicians responded favourably, and we are currently developing training materials that encourage discussion of the clinician-rated measures as part of clinical care [30]. We have also developed a set of training materials that explore the use of the outcome measures as part of the team review process.

We have run numerous training sessions which have made use of the original and more recent training materials, and have offered web-based training. We have presented to around 8,000 people face-to-face and about another 300 via the website. We have directly trained 4,000 clinical staff (including a number who have then acted as local trainers) to use the outcome measures in line with the MH-NOCC protocol. The most recent National Mental Health Report estimates that the public sector clinical workforce comprises around 21,000 full time equivalent (FTE) medical, nursing and diagnostic/allied health staff [31]. Taking into account the fact that our figures represent a head-count (rather than a FTE estimate), we have probably trained at least 20 % of the workforce.

### Optimising the quality of outcome data

Protocol specificity and training consistency were necessary but not sufficient to ensure that Australia’s routinely collected outcome data were of high quality. The vagaries of real-world data collection meant that when we scrutinised the first national dataset in 2004 (which comprised all data from 2001 to 2004), we identified a number of anomalies. It was clear that outcome data were not being collected by all services, or at all of the collection occasions prescribed by the MH-NOCC protocol. Collection occasions could not always be ‘rolled up’ into episodes because they were not always logically sequenced. In addition, individual clinical ratings were not always fully completed.

Over time, we have undertaken a number of activities to improve data quality. In 2004, we prepared a reporting framework [32] to complement the MH- NOCC protocol. This was designed to assist states/territories to monitor the data they received from their local services. It addressed a number of issues around data quality and provided guidance about monitoring adherence with the MH-NOCC protocol.

At the same time, we operationalised the concept of the ‘episode’ in mental health care. Implicit in the MH-NOCC protocol is a series of rules that dictate which sequences of collection occasions legitimately form episodes. For example, an admission followed by a discharge would constitute a legitimate episode whereas two consecutive admissions would not. We identified all of the legitimate combinations of collection occasions, and created an ‘episodiser’ algorithm to automate the process.

We have also tackled the problems of low-volume and incomplete clinical ratings, developing rules for ‘usable’ ratings and exploring ways of dealing with missing data. We have conducted empirical analyses of the completeness of clinical ratings, and communicated the findings (e.g., via jurisdiction-specific presentations / reports, and web-based charts). We have prepared discussion papers related to the comprehensiveness and plausibility of data [33,34], seeking relevant input from experts. We have distributed these through a variety of channels in order to encourage those who are undertaking local analyses to take a similar approach, so that valid comparisons can be made between local and national data.

In order to automate the checking of submitted data, we developed purpose specific software referred to as the ‘validator’. Initially this was used internally by the data bureau in order to process incoming files, and feedback to states/territories (e.g., where data did not comply with the MH-NOCC protocol) was managed manually. A stand-alone ‘validator’ was later developed to allow states/territories to pre-validate the information they were sending. Pre-submission validation has proven indispensable to data quality improvement as it provides appropriate and timely feedback to the submitting staff when they are creating the submission. The success of this approach led to the Department of Health and Ageing commissioning Strategic Data to implement a ‘validator’ for the online submission of three other national mental health minimum data sets (the Mental Health Establishments Collection, National Minimum Data Set for Community Mental Health Care, Residential Mental Health Minimum Data Set). This is now known as the MDS Validator (https://webval.validator.com.au), and supports the validation of incoming data files in terms of their format, structure and plausibility. This service means that data are well regarded by users and is seen by the Department of Health and Ageing and the Australian Institute of Health and Welfare as a model for further improving the efficiency of data acceptance and reporting. We are continuing to develop this tool to ensure that we maximise the extent to which data quality issues can be identified by organisational-level stakeholders.

**Figure 1:**
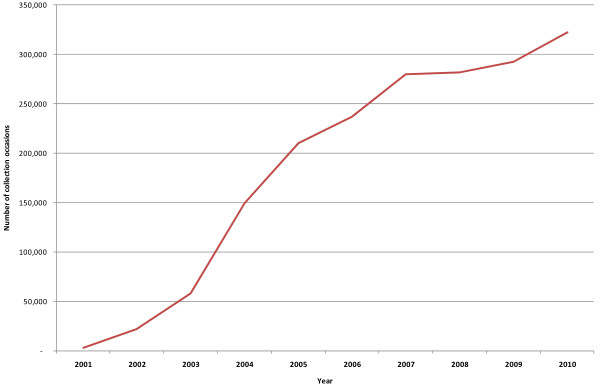
Number of ‘gold’ collection occasions nationally, by year.

These progressive refinements to the data collection and management process have seen an improvement in data quality. We now maintain a ‘gold standard’ data warehouse, which only contains data that comply with the business rules of the MH-NOCC protocol. This not only allows us to work with clean, analysis-ready datasets when we prepare national-level standard reports (see below) but it also provides the most accurate comparison points for clinicians and service managers who are analysing the data for their own local purposes (also see below). Figure 1 shows the total number of ‘gold’ collection occasions by year, and demonstrates that the quality of the data has increased considerably over time. It should be noted that Figure 1 contains data from 2001 to 2010, but 2004 was the first full year when all states/territories began to comprehensively report outcome data.

### Ensuring the clinical utility of outcome data

Stakeholders have consistently commented that clinicians and managers would be more motivated to collect accurate outcome data if these data were useful to them at a local level. For this reason, we have focussed on maximising the clinical utility of outcome measurement.

**Figure 2:**
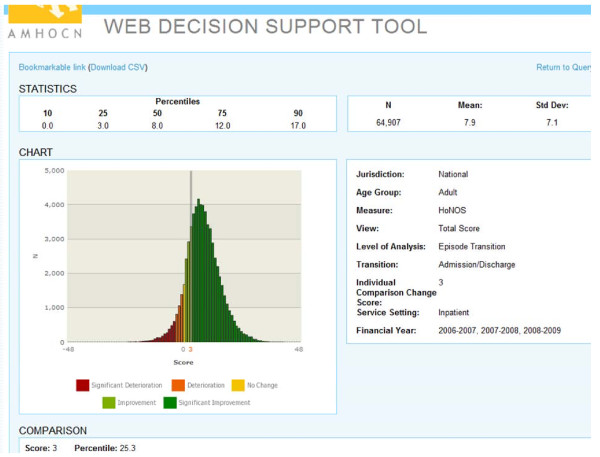
Sample screen shot of the wDST.

As noted above, our ‘second generation’ training materials focused on assisting clinicians to use outcome measurement to inform practice. Alongside these, we have developed various resources for use in the field. For example, in 2006 we developed a Decision Support Tool (DST) which, through data cube technology, enables clinicians to compare their own consumers against normative data from ‘like’ consumers around Australia. The original DST ran on a Microsoft Access platform which was not accessible to all clinicians in all services, so in 2007 we released a web-based version (wDST). Figure 2 provides a sample screen shot for a consumer demonstrating a HoNOS change score of 3, which indicates improvement but only puts him in the 25^th^ percentile compared with the national sample of his peers. The wDST now features in training activities which are designed to introduce clinical staff to the type of information it offers and the potential uses to which this information could be put.

During 2006–07, we undertook what became known as the Clinical Prompts Project. We drew on relevant literature and expert opinion to help us offer junior clinicians suggestions about how they might respond to consumers with particular profiles on various outcome measures. The clinical prompts project culminated in a report published in 2008 [35].

We are currently exploring ways to incorporate the functions of the wDST and the clinical prompts library into services’ local information systems.

### Reporting on outcomes at a national level

We have regularly reported on outcomes at a national level. Our first set of ‘standard reports’ were produced manually in 2005, and included all of the data that had been submitted from 2000–01 to 2004–05 [36]. These reports were partitioned by age group (children/adolescents, adults and older persons) and service setting (inpatient, community residential and ambulatory), and provided a number of different statistical overviews of outcome data (e.g., the change in scores on various measures across the course of given episodes).

Generating these reports was useful in helping us identify data quality issues (see above), and understand some of the issues associated with presenting the data in a meaningful way. However, they were time-consuming to produce and feedback from the field suggested that they were not sufficiently user-friendly. For these reasons, we automated the reporting process, and made the reports more accessible. We developed a ‘reports portal’ which is updated as other developments occur (e.g., refinements to the ‘episodiser’, resolution of particular data quality issues). This allows users to search for particular standard tables via our website. This complements the wDST, described above, which allows users to interrogate the data in more detailed ways.

We have also endeavoured to disseminate national-level outcome data via other channels. For example, we have presented data on the national picture at numerous conferences, and in peer-reviewed journal articles [37].

### Contributing to national and international debates about outcome measurement

We have contributed to debates about outcome measurement, both locally and internationally. One of our early tasks – conducted in 2005 – involved a review of the psychometric properties of the suite of MH-NOCC measures [38,39]. The majority performed well in terms of validity, reliability, sensitivity to change, feasibility and utility. This helped to validate the choice of these measures, but also served to highlight the fact that they were limited to clinician- and consumer-rated measures that were essentially concerned with symptomatology and levels of functioning. Two other reviews followed – one in 2007 and another in 2010. The first considered the potential for introducing a carer measure into the MH-NOCC suite [40], and the second examined the possibility of incorporating measure(s) to assess consumers’ recovery and the recovery orientation of services [41,42]. Both reviews identified existing measures and considered their applicability to the Australian context. We are about to embark on a third review which considers social inclusion measures.

As noted above, we have prepared discussion papers and reports on a range of conceptual and technical issues [43]. We have endeavoured to submit these to rigorous peer review by converting them to academic journal articles wherever possible. Often these publications have been based on our analyses of the most recent national data. For example, we have published extensively on the issues of how best to measure change and which specific indicators might most appropriately be used to measure service effectiveness [44,45], using the national data to demonstrate the consequences of particular approaches. We have also contributed to the practical and theoretical literature on how to ensure ‘like-with-like’ comparisons when using outcome data to compare the effectiveness of services, preparing reports on how to identify ‘peer’ groups of services and how to ‘risk adjust’ for case complexity within services [46]. Sometimes these publications have required us to conduct separate data collection exercises, such as surveys, as in the case of work we undertook to better understand ‘clinical significance’ as it relates to change scores on the HoNOS family of measures [47,48].

### Encouraging the use of outcome data in improving service quality

Throughout our tenure, we have encouraged services to make use of outcome data to improve service quality. Probably the best example of our efforts in this area is our facilitation of the national mental health benchmarking project, which ran from 2006 to 2008 [49-53]. Twenty three organisations from across Australia came together to take part in a number of program-specific (i.e., children and adolescents, adults, older persons and forensic) forums. These forums enabled participating organisations to benchmark themselves against each other, and explore the underlying reasons for their variability in performance against a range of key performance indicators. Participants took part in up to eight face-to-face meetings, and made use of online forums that we developed to facilitate discussion outside of these meetings.

In addition to the benchmarking forums, we have held workshops with broader groups of clinical leaders and service managers from New South Wales and Queensland. These workshops have examined ways in which MH-NOCC and other data can illuminate variations in service provision, with a view to supporting service improvement.

Participants in both the benchmarking forums and the workshops found the examination of intra-organisation variability on particular indicators to be useful in informing service improvements. However, in both cases it was apparent that they needed considerable support, not only to generate the required information but also to interpret it in a meaningful way. Assisting services to develop capacity in ‘information literacy’ is an ongoing focus of our future work in this area.

### Furthering the casemix development agenda

As noted earlier, progress with respect to casemix development has been relatively slow, largely because of our inability to link the MH-NOCC dataset with other relevant datasets. Having said this, we have made some inroads.

In our first foray into casemix development, we circumvented the problem of the inability to link datasets by concentrating on the MH-NOCC dataset in isolation. We focused on inpatient episodes only, and selected those that were anchored by admission and discharge ‘collection occasions’. Using this information, we estimated a length of stay for each episode which we then used as a proxy for resource use. We then examined the performance of two existing casemix classifications to predict length of stay; this was made possible by the fact that the variables that make up these classification systems are included in the MH-NOCC dataset. The first of the two classification systems was developed in the late 1990s for use in the specialist mental health sector, through the Mental Health Classification and Service Costs (MH-CASC) Project [7,54]. The second is the standard classification used in inpatient settings in the general mental health sector, namely the Australian Refined Diagnosis Related Groups (AR-DRGs) [55].

More recently, we have succeeded in linking the MH-NOCC dataset to other relevant datasets. The most recent technical specifications for the MH-NOCC collection require that the same unique identifier be used across the MH-NOCC dataset, the Admitted Patients Mental Health Minimum Data Set, the National Minimum Data Set for Community Mental Health Care, and the Residential Mental Health Minimum Data Set [13]. Using 2008–09 data, we have explored the extent to which there is overlap between consumers identified in the MH-NOCC dataset and consumers identified in the National Minimum Data Set for Community Mental Health Care. Nationally, we found that 30 % of consumers could be identified in both datasets [56]. We are confident that this proportion will increase with time, in the same way that outcome data have improved in quality and comprehensiveness.

## Discussion

When we began our activities, there were few national or international precedents for us to draw upon, either in mental health or in the broader general health sector. Australia was one of the first places to introduce routine outcome measurement in mental health services on such a large scale, although others were beginning to take up the mantle. Notable among these are Ohio in the United States, and New Zealand, both of which have faced the same sorts of issues that we have and (with some contextual nuances) have often addressed them in a similar fashion [57].

We have operated within a framework which has provided an overarching structure to guide our activities, but has also afforded us the flexibility to respond to changing priorities and new issues. We recognised early on that local information systems often required modification and that a significant investment in training was necessary. Our activities have occurred in an iterative fashion, and we have combined a proactive leadership approach with a reactive responsiveness to changing policy priorities and the expressed needs of our various stakeholders. The lack of precedents upon which we could draw has led to a ‘trial and error’ approach; charting new territory has meant that we have often had to modify our activities as we have gone along, in response to feedback from the field. We have often been challenged with the fact that stakeholders have not always been able to tell us what they need until they have had something to respond to. On a related note, we have learnt that it is not reasonable to expect all clinicians and managers to be ‘information literate’ from the outset; we have worked hard to help the mental health sector gain an understanding of how to interpret data in a way that is meaningful and helps to promote service quality.

We have invested considerable effort in progressing the routine outcome measurement agenda over the past seven years, but we are conscious that we still have much to do to capitalise on the current momentum.

· We will need to continue to work with states/territories to improve the quality and utility of data, extending the functionality of the ‘validator’ to undertake further ‘rule checks’. We believe that elevating MH-NOCC to the status of a national minimum dataset would also be desirable; national minimum datasets have particular status in informing benchmarking exercises and in influencing policy, planning and funding decisions.

· We will need to refine our analysis and reporting efforts, so that more refined comparisons can be made between ‘peer’ groups of services.

· We will need to continue to promote the clinical utility of data, examining ways of incorporating the wDST and clinical prompts library into local information systems, and producing online ad hoc reports in response to users’ requests.

· We will need to explore whether there are areas in which the MH-NOCC suite of measures might be strengthened, either by modifying or expanding it (e.g., through the addition of recovery and social inclusion measures) or by reducing it (e.g., by rationalising some of the existing measures). MHISS has endorsed a broader review of MH-NOCC to set directions for the next stage of development beyond the end of the period covered by the Fourth National Mental Health Plan (2009–2014). Our efforts will inform this review.

· In collaboration with the states/territories, we will need to continue to improve the mental health workforce’s ‘information literacy’, and encourage services to benchmark themselves against each other in a manner that allows them to improve their performance.

· We will need to continue the initial and ongoing training of the mental health workforce to minimise ‘rater drift’ and maximise the overall quality of outcome data.

· We will need to continue to promote the public reporting agenda.

· We will need to consolidate our casemix development efforts in the light of broader moves towards activity-based funding, further testing the ability of the MH-CASC and AR-DRG classifications to predict resource use as well as the need to further develop a second generation system. Continuing our data linkage work will be important for this and other purposes.

· We will also need to strengthen our existing strategic partnerships and build new ones, particularly with bodies responsible for promoting and monitoring safety and quality in the broader general health sector.

We believe that our efforts through AMHOCN have contributed to routine outcome measurement gaining a firm foothold in Australia’s public sector mental health services and, that at an average cost to the Australian Government of about $1.9 million per year, we have worked in an efficient manner. These contentions are supported by the findings of HMA’s review, which concluded that ‘AMHOCN has made substantial contributions towards building an information foundation for measuring outcomes and developing mental health casemix concepts in Australia … [Its outputs] represent value for money …’ [58].
